# Genetic hitchhiking can promote the initial spread of strong altruism

**DOI:** 10.1186/1471-2148-8-281

**Published:** 2008-10-10

**Authors:** Mauro Santos, Eörs Szathmáry

**Affiliations:** 1 Departament de Genètica i de Microbiologia, Grup de Biologia Evolutiva (GBE), Universitat Autònoma de Barcelona, 08193 Bellaterra (Barcelona), Spain; 2 Collegium Budapest, Institute for Advanced Study, Szentháromság u. 2, H-1014 Budapest, Hungary; 3 Institute of Biology, Eötvös University, 1/c Pázmány Péter sétány, H-1117 Budapest, Hungary; 4 Parmenides Center for the Study of Thinking, 14a Kardinal Faulhaber Strasse, Munich D-80333, Germany

## Abstract

**Background:**

The evolutionary origin of strong altruism (where the altruist pays an absolute cost in terms of fitness) towards non-kin has never been satisfactorily explained since no mechanism (except genetic drift) seems to be able to overcome the fitness disadvantage of the individual who practiced altruism in the first place.

**Results:**

Here we consider a multilocus, single-generation random group model and demonstrate that with low, but realistic levels of recombination and social heterosis (selecting for allelic diversity within groups) altruism can evolve without invoking kin selection, because sampling effects in the formation of temporary groups and selection for complementary haplotypes generate nonrandom associations between alleles at polymorphic loci.

**Conclusion:**

By letting altruism get off the ground, selection on other genes favourably interferes with the eventual fate of the altruistic trait due to genetic hitchhiking.

## Background

More than thirty years ago Hamilton [1] and Wilson [2] independently discussed the evolution of altruism – cooperative behaviours that decrease the fitness of the individuals that perform them- assuming a structured population divided into small temporary groups; a new view of group selection termed 'trait-group' or 'structured-deme' models [2,3]. A single heritable trait (allele *A*) stimulates its bearer to provide a group benefit, and its eventual spread is conditional on the greater productivity of altruistic groups. Depending on whether or not the recipients of the group benefit include the actor itself, the effects were named 'whole-group' or 'other-only' trait, respectively [4]. Whereas other-only traits are clearly altruistic because involve an absolute cost to the actor, whole-group behaviours generate confusion in the definition of altruism as they may be mutually beneficial to both the actor and the recipients [4,5]. Wilson [3,6] had previously coined the terms 'strong altruism' for those traits involving an absolute cost to the actor (i.e. other-only traits count always as strongly altruistic), and 'weak altruism' when the cost to the actor is only relative.

Contrarily to weakly altruistic traits which can increase in frequency when groups are randomly formed each generation [7], the evolution of strong altruism requires positive assortment for the benefits of altruism to fall preferentially on other altruists [1,6,8,9]. This can be understood in terms of Hamilton's rule [10] of kin selection which states that the condition for an altruistic trait to increase in the next generation is

(1) *rb *> *c*

where *r *is the coefficient of relatedness between actor and recipient, *b *is the fitness benefit (offspring gain) provided to the recipient, and *c *is the reproductive cost to the actor for providing benefits. Under random assortment other group members are a random sample of the global population minus the actor, and relatedness in this case is [4,11,12]

(2) $$r^{o}=\frac{-1}{N-1}$$

(superscript ^*o *^stands for other-only relatedness, and *N *is total population size), which makes it clear that condition (1) cannot be satisfied because kin selection requires a sufficiently high degree of relatedness (*r *> 0) for altruism to evolve. The conclusion that strong altruism does not progress in this model has been recently challenged [13], but the authors assumed multigenerational groups that were initially randomly formed, which just lag the necessary positive assortment among altruists to the next generations. If, however, we stick to Hamilton's [1] original structured-deme model of single-generation, randomly formed groups it still remains undisputed as it has ever been since his formal proof that strong altruism cannot evolve.

No special process has to be invoked to create a structured-deme population as it can simply arise from random distributions of genotypes across patchy resources; a quite common process in many animals, particularly insects. Genetic variation among finite groups creates environmental heterogeneity unrelated to resource heterogeneity [14,15], and there are numerous empirical examples (see especially Table 2 in [16]) showing that there is a positive association between productivity (offspring number) and the levels of genetic variation within a resource. Thus, competition is known to be stronger in 'pure cultures' when compared with genetically diverse groups; and various behaviours may provide a whole-group effect as, e.g., access to resources that are unavailable to solitary individuals, or increased protection to parasites or predators. A recent elaboration on this old theme shows that allelic diversity can be potentially maintained at many loci as it is a positive trait in and on itself [16]. Therefore, differential productivity among groups will not only depend on the frequency of cooperators in a group but also on the level of genetic variation at other loci. Predictions from single-gene theory can be very misleading if it is assumed that in Hamilton's [1] original model haploid individuals are endowed with genomes that code for a range of trait-groups subject to selection, mutation, and recombination. Multilocus systems introduce additional issues such several alternative (meta)stable states [17,18] and the Hill-Robertson effect describing that linkage between loci under selection will reduce the overall effectiveness of selection in finite populations [19,20]. Structured-deme models unavoidably introduce linkage disequilibria that interfere with selection [21], but this effect has never been incorporated in the life cycle interactions that take place between a small number of individuals in a group to study the fate of altruistic traits in a multilocus context.

In this work, we focus on the evolution of altruistic versus selfish alleles and of polymorphisms that show a positive association between productivity and within-group genetic variation to study the fate of altruism in a multilocus context assuming the structured-deme model of single-generation, randomly formed groups originally used by Hamilton [1]. The fate of altruism will be studied by means of computer simulations and mathematical analyses of a relatively simple case. We show that although the altruistic trait is being selected against, when it is genetically linked to productivity enhancing loci altruism can invade. It should be stressed from the very beginning that the model is not producing any assortment mechanism and that it has nothing to do with kin recognition: what happens is that there is a synergy between hitchhiking and group selection that keeps allelic diversity.

## Methods and Results

### Multilocus model and computer simulations

**Figure 1:**
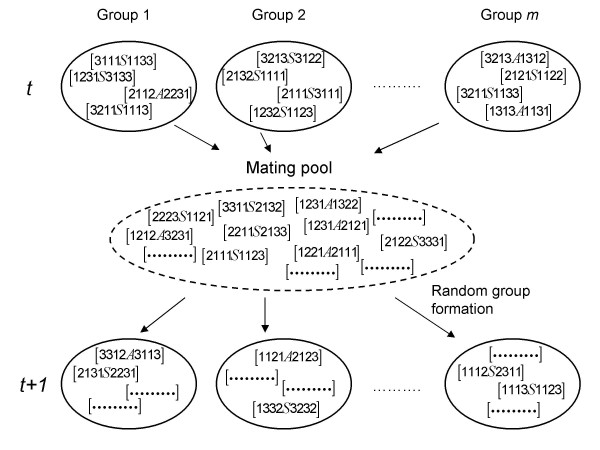
**'Mating pool' mode of reproduction**. A finite population is subdivided into *m *groups. Each group has size *n*. Haploid individuals (represented as [...]) are endowed with *G *loci with 3 alleles each (*j *= 1, 2, 3) that code for whole-group effects according to eq. 3. A focal locus with two alleles (*A*: altruistic; *S*: selfish types) is located in the centre of the set of *G *loci. Selection at the focal locus is modelled following eq. 4. Individuals contribute offspring to a common pool, where random pairing of two haplotypes and recombination between adjacent loci with frequency *R *occurs. Haploid individuals again form new groups by random assortment. Notice that at the focal locus we are dealing with the classical group selection model analyzed by Hamilton [1].

Figure 1 illustrates the 'mating pool' mode of reproduction on which our model is based. A haploid finite population of size *N *= *m *× *n *is randomly subdivided into *m *groups with *n *individuals per group. Offspring genotypes at each generation are sampled from a common population and randomly subdivided into temporary groups where individuals representing a finite sample from the pooled distribution reproduce proportional to their fitness. Productivity enhancing (offspring number) genes when in genetically diverse groups coded by *g *= 1, ⋯, *G *loci were stored as lists on a single chromosome. The haploid population was initially polymorphic for *i *= 3 alleles at equal frequencies. With random grouping the distributions of the different compositions of groups for each of the *i *alleles will be binomial with parameters (*p*_*i*_, *n*), where *n *is the group size. Assuming that allele 1 is the fittest one, within-group selection at the gth locus was modelled as

(3) $$\begin{array}{l} \text{Homogeneous groups at }\\ \text{locus }g\;\left\{ \begin{array}{l} W_{1}^{g}=1\\ W_{j\left(j\neq 1\right)}^{g}=f_{s}\text{; }f_{s}\leq 1\text{;} \end{array}\right.\\ \text{Heterogeneous groups at }\\ \text{locus }g\;\left\{ \begin{array}{l} W_{1\cup j}^{g}=1+y\left(a^{z}-1\right)\\ W_{j\cup k}^{g}=f_{d}\left[1+y\left(a^{z}-1\right)\right]\text{; }\forall k\neq j,f_{d}\leq 1 \end{array}\right. \end{array}$$

where *y *is a constant for the proportional increase of group's fitness as a function of allele diversity *a *(i.e. number of alleles in a group); and *z *models the benefits of allele diversity upon fitness. Therefore, without genetic diversity increasing group's fitness directional selection drives allele 1 to fixation whenever *f*_*s *_or *f*_*d *_<1. We set *y *= 0.5, *z *= 1, and *f*_*s *_= *f*_*d *_= 0.95. Allelic diversity can easily persist in this model under a wide range of parameter values [16,21], and there are up to 3^*G *^possible segregating haplotypes.

After an initial period of 500 generations to allow the population to reach a quasi-equilibrium state under selection and free recombination between adjacent loci, the focal strongly altruistic locus was introduced in the centre of the set of those whole-group beneficial loci with alleles sampled from a binomial distribution with *p *= 0.05 and *q *= 0.95 for the *A*- and *S*-types, respectively. Selection at the focal locus was modelled as in Hamilton's [1] original model

(4) $$\begin{array}{l} W_{A}=1-k+\left(v-1\right)\frac{K}{n-1}\text{;}\\ W_{S}=1+v\frac{K}{n-1}, \end{array}$$

where *v *is the number of altruistic members in a group of size *n*, and *k *is the units of fitness given by an *A*-type in order to add *K *units to the joint fitness of his (*n *-1) companions. The degree of positive assortment (*F*) required for altruism to evolve is [1]

(5) $$\frac{K}{k}=\frac{1}{F}.$$

In other words, the critical *c/b *ratio for positive selection of altruism is *k*/*K*.

With *N *= 240 and n = 4 as assumed in most simulations (but see below) only 60 groups are formed, and the hypergeometric probability (i.e., sampling is without replacement) of randomly forming homogeneous groups of altruists with initial *p *= 0.05 is 3.67 × 10^-6^. Random group formation events will result in relatedness (*r*^*o*^) values which fluctuate around – 1/(*N- *1) for the altruistic locus. With *k *= 0.1 and *K *= 0.2 as assumed here strong altruism is expected to be lost a few generations after its introduction into the population since homogeneous groups of altruists will be surely absent.

With multiple loci the probability of allele fixation increases in the whole-group beneficial loci [21], and mutation was introduced whenever a locus *g *was fixed for one allele. This is, however, a relatively minor problem with less than *G *= 40 loci or so [21], and the only point to introducing mutation was to keep constant the 'effective' number of segregating *G *loci in the population.

We simulated repeated introductions of the *A *allele into the population whenever the *S*-type reached fixation. The lists of loci could undergo a recombination process following a stochastic multilocus method [22] with recombination frequency between adjacent loci denoted by *R *(no interference was assumed). In each generation, the sequence of events was mating pool formation, mutation, selection, recombination, and subdivision. We assumed multiplicativity of selective effects. Roulette selection operator, in which the chance of a chromosome getting selected is proportional to its fitness, was used. Selected chromosomes were randomly paired for recombination and randomly assigned to a group. Up to seven computer-simulation trials with different random number seeds were run for each combination of parameter values, and each trial was run for up to 50,000 generations. By following the fate of the *A*-type variant introduced repeatedly into the population, until it is fixed or lost, we can estimate the mean times to fixation or loss, respectively. The simulations were implemented in MATLAB version 7.2 [23]. An analytical treatment of the model in a simplified situation with group size *n *= 2 and no recombination is given after the numerical results.

### Numerical results

In order to numerically demonstrate how strong altruism can evolve in single-generation, randomly formed groups, consider a focal locus with two alleles (*A*: altruistic; *S*: selfish types) embedded in a multilocus genome containing *g *= 1, ⋯, *G *selected loci stored as lists on a single chromosome with whole-group beneficial traits in quasi-equilibrium state under selection (see above). Group members interact for one generation and because of behaviours individuals perform during such single-generation associations, group members affect each other fitness and may also enjoy certain individual-level benefits expressed by groups composed of genetically different individuals before the population is pooled back again and randomly forms new groups in the next generation. We have chosen this model primarily because it explicitly incorporates the key characteristics of the Hill-Robertson effect – random sampling and selection at multiple (independent) loci. Here we explore the extent to which recombination influences the evolution of an altruistic allele surrounded by other loci subject to selection.

**Figure 2:**
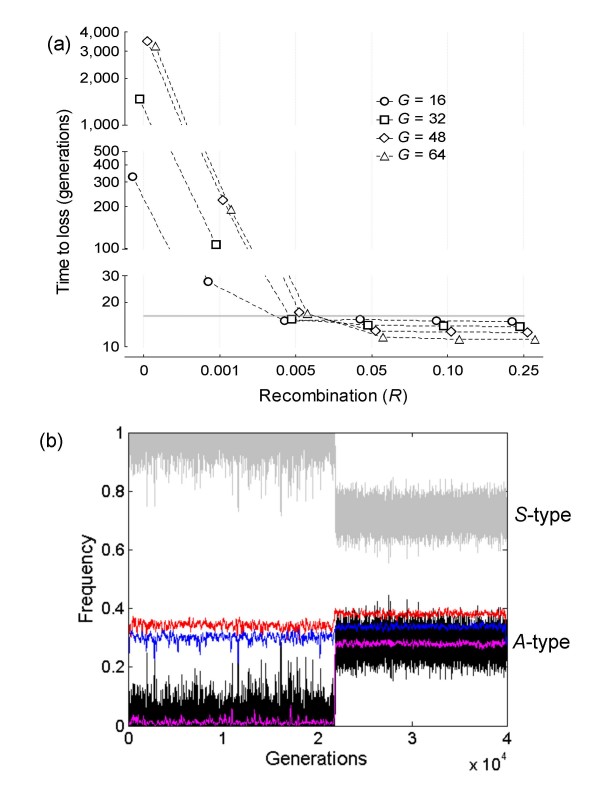
**Multilocus trait-group model**. **(a) **Semi-log plot for the mean time to loss of the strongly altruistic trait coded by allele *A *as a function of *R *estimated from independent simulation trials with up to 50,000 generations each. Population size was kept constant with *n *= 4 and *m *= 60. Allele *A *was surrounded by *G *= 16, 32, 48, 64 loci. The dotted horizontal line at generation 16 indicates the mean persistence time of allele *A *for the single gene situation, where the total number of introductions in a typical trial was > 3, 000 and the maximum number of generations before allele *A *was lost ~100. When surrounded by the *G *loci, the number of introductions of the *A *allele into the population was obviously dependent on its ultimate fate, ranging from 43 (*R *= 0) to > 20, 000 (*R *≥ 0.10) when all trials for a given set of *G *loci are considered. **(b) **Sample simulation with *G *= 8 and *R *= 0 that illustrates the eventual locking in quasi-equilibrium state of the *A*-type due to complementary segregating haplotypes. After introduction 1,532 at generation 21,843 the *A*-type (dark black jagged line pattern) remained segregating in the population, whose genotypic composition consisted of three haplotypes: [1211*S*2112] in red at average frequency 0.3817, [1322*S*3121] in blue at 0.3382, and [2133*A*1313] in magenta at 0.2802 (equilibrium frequency of the *A*-type). To enhance visibility the haplotype line patterns were smoothed by using a moving average of 100 generations.

The population size was fixed to *N *= 240 individuals. This introduces high stochasticity (but see below for the effect of both population size and group size), but parameters in the *c/b *ratio were chosen as to make it very unlikely that the *A*-type could be fixed by genetic drift in the single locus case (ultimate loss of the altruistic allele was constantly observed after > 2.5 × 10^5 ^introductions into the population with *n *= 4). An initial survey of a range of number of loci and values for the proportional increase of group's fitness as a function of allele diversity at locus *g *was made, and here we present summary results with group size *n *= 4; *G *= 16, 32, 48, 64 loci. As expected, the numerical outcomes show that the mean persistence time of the altruistic *A*-type is influenced by recombination between adjacent linked loci (Figure 2a). When embedded in a chromosome, or chromosome fragment, with tight linkage *R *≤ 0.001 (notice that an *R *value of 10^-3 ^between adjacent loci would correspond to a region approximately (*G *+ 1)× 100 × 10^-3 ^centimorgans in length and roughly equivalent to a chromosome with 1,000 genes and total map distance 100 centimorgans), the altruistic allele could easily persist in the population for quite a long time because the genome eventually crystallized in a few segregating haplotypes. This effect was already clear with only *G *= 8 loci, a situation that allows tracking the haplotype dynamics and the eventual trapping of the *A*-type by complementary segregating haplotypes (Figure 2b).

The results can be understood as follows. Deterministic haplotype dynamics assuming linkage equilibrium at generation *t*_0 _guarantees that any *A*-type mutant introduced into the population will be damned to eventual loss no matter how many *G *loci are segregating in the population (see analytical treatment below). Variance in linkage disequilibrium is generated directly by random drift, and the *A*-type can be kept in the population when statistically associated (hitchhike) with haplotypes that remain segregating at quasi-equilibrium state. Stochasticity in this model is obviously a function of haplotype space that a given population size can explore. Since there are up to 3^*G *^possible segregating haplotypes for the *G *loci, no real population will eventually behave according to the deterministic dynamics and hitchhiking effects related to the action of social heterosis may prevent the altruistic allele from going quickly extinct. With low recombination neighbouring genes tend to be inherited together, and those three segregating haplotypes in the sampled simulation (Figure 2b) can be thought of as alleles of a whole-group effect supergene, with all group members experiencing a net benefit from genetic diversity. Relatedness in this case is [13]* r*^*w *^= 1/*n *(superscript ^*w *^stands for whole-group relatedness) and is obviously positive. This does not, however, invalidate our claim that strong altruism can invade without kin selection because relatedness at the focal locus still remains *r*^*o *^= -1/(*N*-1), so there is no kin selection effect involved. The role of group size *n *can also be easily visualized. A large group size will tend to have low across-group variance in genetic diversity, which eventually drives haplotype [1211*S*2112] in Figure 2b to fixation since we have assumed that at each *g*th locus allele 1 is the fittest one (eq. 3).

**Figure 3:**
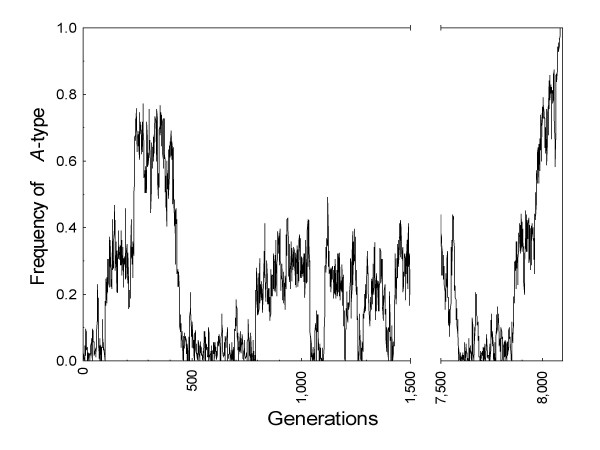
**Fixation of strong altruism in Hamilton's random group model**. With *G *= 64 loci and *R *= 0.005 the probability of fixation of the strongly altruistic *A*-type estimated after 8,652 independent introductions was 5.78 × 10^-4^. The plot shows a sample simulation where the *A*-type reached fixation at generation 8,088 after 330 prior unsuccessful introductions into the population. Time to fixation was 241 generations.

Recombination breaks down the statistical associations between alleles at linked sites (i.e. detaches *r*^*o *^from *r*^*w*^) but, as we might expect, the hitchhiking effect is most marked when there are many segregating loci. With intermediate linkage (*R *= 0.005) fixation of strong altruism was a sporadic outcome for *G *= 32 and *G *= 48 loci, and happened with a relatively high probability for *G *= 64 loci (Figure 3). In these cases it was computationally unfeasible to keep track of all segregating haplotypes in the population, but the results clearly suggest that the eventually successful *A*-allele was locked around a haplotype region that went to fixation or, alternatively, to various regions that happened to reach a high enough frequency (see below). Finally, swift loss of the *A*-type was always the final outcome with *R *≥ 0.10.

### Analytical dynamics of whole-group genes

In order to understand the genetic hitchhiking effect responsible for the spread of strong altruism in our group selection model, it is important to first understand the dynamics of whole-group genes since linkage disequilibria can also be generated by deterministic forces. To clarify matters, we deal here with the simplest situation where the *g *= 1, ⋯, *G *loci with whole-group beneficial traits have two alleles each (e.g., 1^1 ^and 2^1^; 1^2 ^and 2^2 ^for a 2-locus genome). For each pair, allele 1 is the fittest one as assumed in Methods. This creates up to 2^*G *^possible haplotypes. We set group size to *n *= 2 and assume no recombination (*R *= 0). It is then possible to easily follow the dynamics of all segregating haplotypes in the population through time. Assuming that the benefits of allele diversity upon fitness are linear (*z *= 1 in eq. 3), the payoff matrix for the *g*th locus is

(6) $$P^{g}\text{: }\left|\begin{array}{ccc} & 1^{g} & 2^{g}\\ 1^{g} & w_{11}=1 & w_{12}=\left(1+y\right)\\ 2^{g} & w_{21}=f_{d}\left(1+y\right) & w_{22}=f_{s} \end{array}\right|$$

where element *w*_*ij *_is the fitness of individual *i *(1^*g*^, 2^*g*) ^in a group with individual *j*. With *G *loci and multiplicativity of selective effects the payoff matrix is simply

(7) **Γ **= **P**^1 ^⊗ **P**^2 ^⊗ ⋯ ⊗**P**^*G*^

where ⊗ is the Kronecker tensor product. For *G *= 2 this gives the matrix

(8) $$\begin{array}{cc} \Gamma \text{:} & \left|\begin{array}{ccccc} & 1^{1}1^{2} & 1^{1}2^{2} & 2^{1}1^{2} & 2^{1}2^{2}\\ 1^{1}1^{2} & 1 & \left(1+y\right) & \left(1+y\right) & {\left(1+y\right)}^{2}\\ 1^{1}2^{2} & f_{d}\left(1+y\right) & f_{s} & f_{d}{\left(1+y\right)}^{2} & f_{s}\left(1+y\right)\\ 2^{1}1^{2} & f_{d}\left(1+y\right) & f_{d}{\left(1+y\right)}^{2} & f_{s} & f_{s}\left(1+y\right)\\ 2^{1}2^{2} & f_{d}^{2}{\left(1+y\right)}^{2} & f_{s}f_{d}\left(1+y\right) & f_{s}f_{d}\left(1+y\right) & f_{s}^{2} \end{array}\right| \end{array}$$

It is clear that group mean is

(9) $$\Psi =\frac{1}{2}\left(\Gamma +\Gamma^{T}\right)$$

where superscript ^*T *^signifies matrix transposition. Now we have to generate the appropriate matrix of haplotypes, which can be done as follows. At generation *t*_0 _segregating alleles have frequencies $p_{0}^{g}$ and 1 - $p_{0}^{g}$ for locus *g*, and linkage disequilibrium is absent (*D *= 0). Assume for simplicity *G *= 2 loci; then the 4 × 4 haplotype matrix is obtained in two steps. First, we multiply the allele frequency vectors to obtain the haplotype frequencies

(10) $$h=\left[p_{0}^{1}\;\left(1-p_{0}^{1}\right)\right]⊗\left[p_{0}^{2}\;\left(1-p_{0}^{2}\right)\right],$$

and second, we multiply the resulting **h **vector

(11) **v **= **h **⊗ **h**

**Figure 4:**
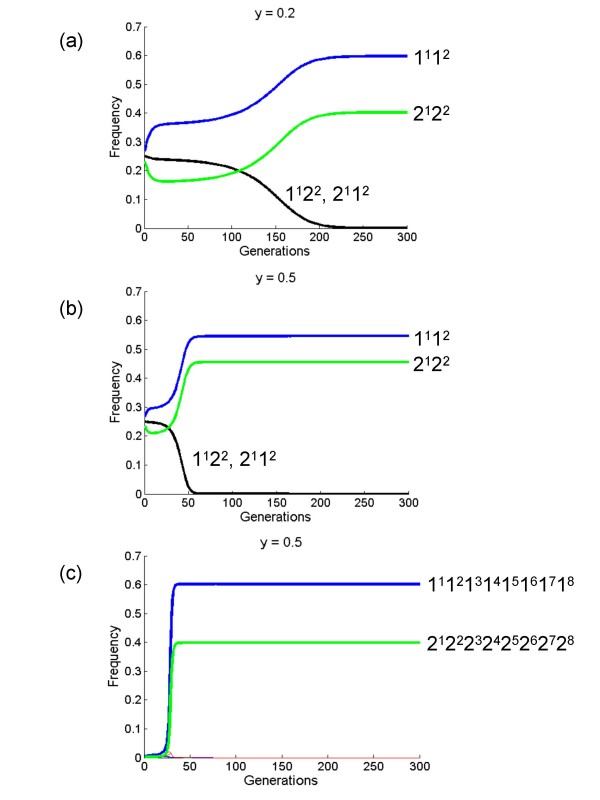
**Deterministic haplotype dynamics for the whole-group loci without recombination**. At generation *t*_0 _all segregating alleles were assumed to be at equal frequencies, and linkage disequilibrium was absent. (a), (b), Two-loci haplotypes, with *y *values as given at the top of each panel and haplotypes as indicated. (c), Eight-loci haplotypes.

This row vector can now be rearranged by noting that its first 2^*G *^elements are the corresponding random group frequencies of haplotype 1^1^1^2 ^in a group with haplotype 1^1^1^2^, ⋯, 2^1^2^2^; the second 2^*G *^elements the corresponding frequencies of haplotype1^1^2^2 ^in a group with haplotype 1^1^1^2^, ⋯, 2^1^2^2^; etc. After rearranging this vector we obtain the suitable matrix of haplotypes (**H**). Since *R *= 0, the recurrence relations for the haplotype frequencies are

(12) $$\bar{w}h_{i}=\sum_{i}H_{i}⊙\Gamma_{i}⊙\Psi_{i},$$

where

$$\bar{w}=\sum_{i,j}H_{i,j}⊙\Gamma_{i,j}⊙\Psi_{i,j}$$

is the average fitness, *h*_*i *_is the corresponding row for the *i*th haplotype (individual), and ⊙ denotes element-by-element multiplication of the corresponding *i*, *j *elements in the three matrices (also known as a Hadamard product). Letting *f*_*s *_= *f*_*d *_= 0.95 in payoff matrix (6), Figure 4 shows the haplotype dynamics for different values of the proportional increase of group's fitness *y *as a function of allele diversity.

It is clear that with the assumed starting conditions the deterministic short-term evolution drives the population to end up with only coupling haplotypes at equilibrium state. We can also define ESS (Evolutionary Stable Strategy) conditions for the payoff matrix (8) by noting that the game theoretical model is isomorphic to a trait group model with group size *n *= 2. Inspection of (8), by application of standard game theory [24] and dynamics [25], reveals that there are only two ESSs, corresponding to complete coupling {1^1^1^2^, 2^1^2^2^} and repulsion {1^1^2^2^, 2^1^1^2^} haplotypes. All other equilibria are unstable. This holds if *f*_*s*_, *f*_*d*_, *y *< 1; *y *> (1- *f*_*d*_)/*f*_*d*_. The domains of attraction of the two ESSs are not equal, hence from initial linkage equilibrium the system converges to the coupling ESS (Figure 4). It is straightforward to show that by increasing the number of loci by one, the number of mixed ESSs doubles, i.e. the latter scales with 2^*G*-1 ^if the number of alleles per locus is 2. These considerations clearly suggest that there is a balance between recombination (always decreases linkage disequilibrium) and selection (convergence to coupling or repulsion haplotypes) in the system.

The preceding analysis can explain some previous numerical results [26]. Computer trials using bi-allelic loci with all haplotypes initially at equal frequencies and finite population size *N *= 120, with group size set to *n *= 2 and parameter values *f*_*s *_= *f*_*d *_= 0.95 and *y *= 0.2, had found that coupling haplotypes were the most common ones left in the population assuming no recombination, but in 42.8% of the trials with *G *= 2 loci, and 56.5% with *G *= 3 loci, the fittest haplotype was lost from the population. There was, however, strong selection for allelic diversity at each locus to be maintained. This was most evident when repulsion haplotypes were the only ones left, as they remained segregating in the population at equilibrium state. Finally, it should be stated here that by letting *f*_*d *_= 1 in payoff matrix (6) the system converges to the standard heterotic case [17,18,27].

### Fate of the altruistic allele in the analytical dynamics

It is straightforward to incorporate the altruistic locus in the former analytical treatment for the whole-group genes. The payoff matrix is now (eq. 4)

(13) $$P^{a}\text{: }\left|\begin{array}{ccc} & S & A\\ S & 1 & \left(1+K\right)\\ A & \left(1-k\right) & \left(1-k+K\right) \end{array}\right|$$

where *S *and *A *stand for the selfish and (strongly) altruistic types, respectively; and *k/K *is the critical ratio for positive selection of altruism. With multiplicativity of selective effects and *G *whole-group loci the multilocus payoff matrix is

(14) **Γ **= **P**^*a *^⊗ **P**^1 ^⊗ ⋯ ⊗ **P**^*G*^

which, with *G *= 1 becomes

(15) $$\begin{array}{cc} \Gamma \text{:} & |\begin{array}{ccc} & S1^{1} & S2^{1}\\ S1^{1} & 1 & \left(1+y\right)\\ S2^{1} & f_{d}\left(1+y\right) & f_{s}\\ A1^{1} & \left(1-k\right) & \left(1-k\right)\left(1+y\right)\\ A2^{1} & \left(1-k\right)f_{d}\left(1+y\right) & f_{s}\left(1-k\right) \end{array}\;\cdots \\ & \begin{array}{ccc} & A1^{1} & A2^{1}\\ S1^{1} & \left(1+K\right) & \left(1+K\right)\left(1+y\right)\\ S2^{1} & \left(1+K\right)f_{d}\left(1+y\right) & f_{s}\left(1+K\right)\\ A1^{1} & \left(1-k+K\right) & \left(1-k+K\right)\left(1+y\right)\\ A2^{1} & \left(1-k+K\right)f_{d}\left(1+y\right) & f_{s}\left(1-k+K\right) \end{array}| \end{array}$$

It is easy to see that {S1^1^, S2^1^} is the only (mixed) ESS, thus selfishness prevails. With *G *≥ 2 the problem is also straightforward. If the altruistic allele is fixed in the population, but otherwise all haplotypes are present, then the metastable equilibria exactly mirror those of the system without the altruistic locus, i.e. for *G *= 2 loci the equilibria are the same as for matrix (8). Suppose now that the system is in the equilibrium {*A*1^1^1^2^, *A*2^1^2^2^}. Which haplotypes can invade the population? Inspection of the **Γ **matrix (15) reveals that only haplotypes *S*1^1^1^2 ^and *S*2^1^2^2 ^can invade, but *S*1^1^2^2 ^and S2^1^1^2 ^cannot, *even though the latter also carry the selfish allele*, provided *f*_*d*_>(1 + *K*)/[1- *k *+ *K*)(1 + *y*)]. This means that the pair {*A*1^1^1^2^, A2^1^2^2^} is unstable *in the direction of the same heterotic haplotypes only*, carrying the selfish allele. This generalizes to more loci in an important way. As the number of loci increases, it remains true that the resident, altruistic pair of haplotypes can be invaded by two appropriate haplotypes only; all the other selfish haplotypes are repelled.

Deterministic haplotype dynamics assuming linkage equilibrium at generation *t*_0 _also shows that any *A*-type mutant introduced into the population is damned to eventual loss no matter how many *G *loci are segregating in the population. The only possibility for the *A*-type to be kept in the population is to statistically associate itself (hitchhike) with haplotypes that remain segregating at quasi-equilibrium state.

In finite populations allelic diversity can be lost when the fittest haplotype is present since its eventual fixation can occur [26], a result which strongly depends on the number of *G *loci initially introduced in the population and the strength of selection [21]. This can be understood by comparing Figures 4b and 4c, where the equilibrium frequency of the fittest haplotype is higher as the number of loci increase. Because in the model for whole-group genes there is also strong selection to maintain allelic diversity it is, therefore, not surprising that a mutant *A*-type could eventually hitchhike a haplotype that is complementary to those already present in the population. The interaction between linkage, selection, and sampling in structured-deme models is precisely the phenomenon that can be described by the 'Hill-Robertson' effect [19] or genetic hitchhiking [20].

We can now envisage the effects of group size *n *and population size *N *on the eventual fate of allele *A *under a given set of conditions. Increasing group size decreases the across-group variance in allelic diversity at the *G *loci, which in turns increases the within-group selection and reduces the likelihood of polymorphism since the fittest allele *1*^*g *^at the *g*th locus goes to fixation (eqs. 3 and 6). Hence, hitchhiking may not be possible and the eventual dynamics of the *A*-type converges to the single locus case. In other words, the initial spread of altruism in this model is conditional on a large enough among-group genetic variance and will not happen in genetically homogeneous settings. Simulations with *N *= 240, *G *≥ 16, and parameter values as above indicate that group size *n *≥ 16 is large enough as to dramatically reduce or even prevent the eventual trapping of the *A*-type by complementary segregating haplotypes when *R *= 0.

The effect of population size *N *is relatively more complex since stochasticity in this model is obviously a function of the strength of selection at each locus and the haplotype space that a given population can explore. For instance, in simulations with *G *= 8 (haplotype space 2 × 3^8 ^= 13, 122; including the focal altruistic locus), *n *= 4, *R *= 0 and strong selection quasi-stable polymorphism of the *A*-type was not detected when *N *≥ 500 since haplotype evolution tends to approach the deterministic situation; but was a frequent outcome with *G *≥ 32 loci even when *N *= 2, 000. On the other hand, reducing the strength of selection by 60% (i.e., *y *= 0.2, *k *= 0.04 and *K *= 0.08) allowed to increase population size to *N *= 500 as to qualitatively obtain similar results for *G *= 8 as those obtained with strong selection and *N *= 240. In summary, the hitchhiking effect is robust for large population sizes as long as group size *n *is small enough, so that among-group allelic diversity at the *G *loci can be selectively kept.

**Figure 5:**
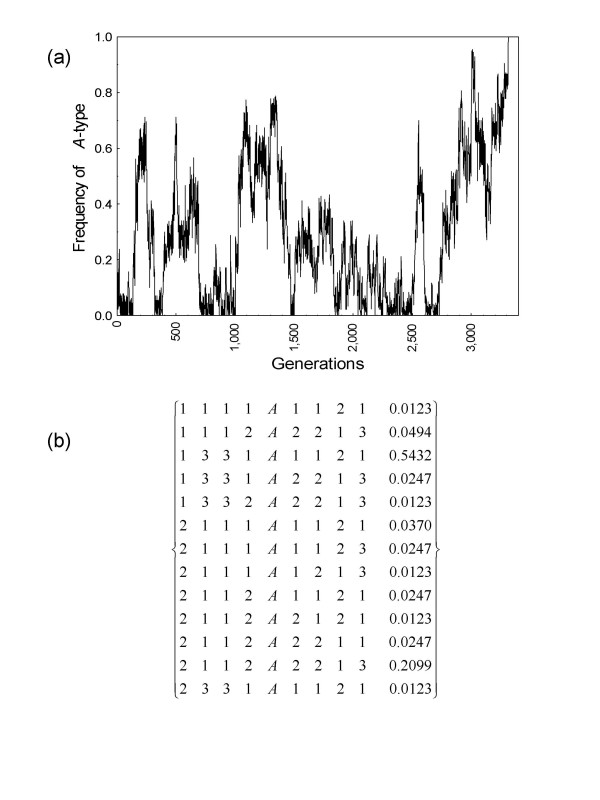
**Snapshot of segregating haplotypes after fixation of *A *allele**. **(a)**, Sample simulation with *G *= 64 loci and *R *= 0.005 showing the fixation of the strongly altruistic *A*-type at generation 3,315 after 133 prior unsuccessful introductions into the population. **(b)**, Snapshot of 13 segregating haplotypes around the focal locus out of 81 haplotypes when all loci are considered. Frequencies are given in the last column. The two most frequent haplotypes are clearly complementary.

In the simulations with *G *= 64 loci and *R *= 0.005 we detected a relatively high probability of fixation of strong altruism (5.78 × 10^-4^). Figure 5 also shows a sample simulation where a snapshot of segregating haplotypes right after fixation of the *A*-type was obtained. As expected from the analytical treatment above, it is clear that the successful *A*-allele was locked around a complementary region that reached a high enough frequency because it allowed group members to experience a net benefit from genetic diversity at the *G *loci.

## Discussion and Conclusion

We acknowledge that our structured-deme model assumes strong selection in the collections of individuals who influence one another's fitness, mainly to prevent waiting times before the eventual spread of the altruistic trait to becoming computationally unmanageable. The important point, however, is to understand that linkage, selection, and sampling will necessarily interact in multilocus structured-deme models, and the interference effect will extend to all loci in a block depending on the recombination value. With tight linkage a few selected loci may be enough to trap the altruistic allele in a quasi-equilibrium polymorphic state. With moderate recombination fixation of strong altruism can occur. Our results do pose a serious challenge to the universally accepted view that kin selection is the key component to explain altruistic behaviours that impose an absolute fitness cost to the actor and, to some extent, might be relevant to understand the origin (as opposed to the maintenance) of eusociality [28,29]. No matter how stable can the long-term evolution of altruism be (i.e., resident altruistic haplotypes can always be invaded by appropriate, and only the appropriate, haplotypes carrying the selfish allele; see above), the results suggest that genetic hitchhiking is a basic ingredient for the evolution of cooperation considering the crucial problem faced by altruistic behaviours in structured-deme models: their initial establishment when rare [30]. As forcefully stressed by Field [31], we should avoid here the 'inverse genetic fallacy'; namely, the inappropriate attribution of mechanisms that may be sustaining cooperation to the explanation of its origin. This is clearly the case here when contrasting with the consequences that positive assortment (i.e., altruists settle with altruists) can have on the maintenance of altruism.

Positive assortment increases the relatedness at the focal altruistic locus and, depending on the linkage disequilibrium between that locus and nearby *G *loci it also increases the relatedness at those loci. This, in turn, decreases the within-group variance and the likelihood of genetic hitchhiking since the fittest allele at the *g*th whole-group locus can become fixed more easily. We thus have a powerful mechanism to explain the evolutionary origin of altruism, but unless linkage disequilibria tend to be reduced its long-term maintenance (by kin selection) can be compromised if group-beneficial effects exerted by social heterosis and random grouping are stronger than those exerted by the focal locus under positive assortment. As long as the formation of groups remains random, there is no chance to cast our results in terms of kin selection: thus, kin selection and social heterosis are in this sense opposing mechanisms to help the initial spread of strong altruism. We are not considering the chance spread of just any maladaptive allele; rather, the necessary synergy between hitchhiking and (strong) group selection that keeps allelic diversity.

In the same vein that multivariate selection theory provides a framework to predict the direct and indirect effects of selection on a suite of complex traits [32] and illustrates the empirical shortfalls of focusing on a single character, we hypothesize that multilocus approaches to social evolution [33] will eventually demonstrate that cooperation can evolve without assuming interactions between relatives: the condition will be that indirect effects of selection on linked loci should be greater than the direct response to selection at the focal altruistic locus. Even if Hamilton's condition (1) applies, the effects discussed in this paper may help the initial spread of altruists.

## Authors' contributions

Both authors contributed equally to this manuscript. Both authors read and approved the final manuscript.
